# Supplementary material for: Modeling glioblastoma heterogeneity as a dynamic network of cell states
Source: Mol Syst Biol. 2021 Sep 16;17(9):e10105. doi: 10.15252/msb.202010105 (PMC8444284; doi:10.15252/msb.202010105)
Supplement: Supplementary file 5 — Source Data for Figure 3 [file MSB-17-e10105-s001.zip › Figure3A_sourcedata/GSEA_3065/hallmarks_state3.GseaPreranked.1623416274744/gsea_report_for_na_neg_1623416274744.html]

Report for na\_neg 1623416274744 [GSEA]

| GS  follow link to MSigDB | GS DETAILS | SIZE | ES | NES | NOM p-val | FDR q-val | FWER p-val | RANK AT MAX | LEADING EDGE || 1 | HALLMARK\_OXIDATIVE\_PHOSPHORYLATION | Details ... | 184 | -0.66 | -2.56 | 0.000 | 0.000 | 0.000 | 1220 | tags=46%, list=14%, signal=53% |
| 2 | HALLMARK\_MYC\_TARGETS\_V1 | Details ... | 190 | -0.50 | -1.95 | 0.000 | 0.000 | 0.001 | 1290 | tags=36%, list=15%, signal=41% |
| 3 | HALLMARK\_DNA\_REPAIR | Details ... | 123 | -0.52 | -1.91 | 0.000 | 0.000 | 0.001 | 1440 | tags=40%, list=16%, signal=47% |
| 4 | HALLMARK\_REACTIVE\_OXYGEN\_SPECIES\_PATHWAY | Details ... | 36 | -0.63 | -1.87 | 0.000 | 0.001 | 0.004 | 490 | tags=28%, list=6%, signal=29% |
| 5 | HALLMARK\_PI3K\_AKT\_MTOR\_SIGNALING | Details ... | 80 | -0.45 | -1.59 | 0.003 | 0.020 | 0.178 | 1329 | tags=29%, list=15%, signal=34% |
| 6 | HALLMARK\_P53\_PATHWAY | Details ... | 132 | -0.41 | -1.51 | 0.006 | 0.037 | 0.359 | 915 | tags=19%, list=10%, signal=21% |
| 7 | HALLMARK\_ADIPOGENESIS | Details ... | 150 | -0.34 | -1.30 | 0.049 | 0.180 | 0.922 | 1203 | tags=25%, list=14%, signal=28% |
| 8 | HALLMARK\_TGF\_BETA\_SIGNALING | Details ... | 45 | -0.38 | -1.22 | 0.171 | 0.283 | 0.992 | 819 | tags=20%, list=9%, signal=22% |
| 9 | HALLMARK\_FATTY\_ACID\_METABOLISM | Details ... | 102 | -0.34 | -1.21 | 0.157 | 0.270 | 0.994 | 1098 | tags=19%, list=13%, signal=21% |
| 10 | HALLMARK\_PEROXISOME | Details ... | 64 | -0.32 | -1.06 | 0.356 | 0.557 | 1.000 | 1098 | tags=22%, list=13%, signal=25% |
| 11 | HALLMARK\_TNFA\_SIGNALING\_VIA\_NFKB | Details ... | 101 | -0.30 | -1.05 | 0.370 | 0.526 | 1.000 | 2171 | tags=33%, list=25%, signal=43% |
| 12 | HALLMARK\_HEME\_METABOLISM | Details ... | 117 | -0.25 | -0.93 | 0.606 | 0.786 | 1.000 | 1612 | tags=23%, list=18%, signal=28% |
| 13 | HALLMARK\_HYPOXIA | Details ... | 122 | -0.25 | -0.92 | 0.627 | 0.756 | 1.000 | 1835 | tags=26%, list=21%, signal=33% |
| 14 | HALLMARK\_INTERFERON\_GAMMA\_RESPONSE | Details ... | 94 | -0.24 | -0.86 | 0.744 | 0.844 | 1.000 | 1369 | tags=16%, list=16%, signal=19% |
| 15 | HALLMARK\_KRAS\_SIGNALING\_DN | Details ... | 31 | -0.23 | -0.67 | 0.917 | 1.000 | 1.000 | 1978 | tags=29%, list=23%, signal=37% |
| 16 | HALLMARK\_MYC\_TARGETS\_V2 | Details ... | 46 | -0.20 | -0.62 | 0.960 | 0.989 | 1.000 | 1528 | tags=15%, list=17%, signal=18% |
Table: Gene sets enriched in phenotype **na **[plain text format]****
